# Elevated levels of 2-arachidonoylglycerol promote atherogenesis in ApoE^-/-^ mice

**DOI:** 10.1371/journal.pone.0197751

**Published:** 2018-05-29

**Authors:** Julian Jehle, Benedikt Schöne, Sayeh Bagheri, Elina Avraamidou, Melina Danisch, Imke Frank, Philipp Pfeifer, Laura Bindila, Beat Lutz, Dieter Lütjohann, Andreas Zimmer, Georg Nickenig

**Affiliations:** 1 Department of Internal Medicine II Cardiology, Pneumology, Angiology, University Hospital Bonn, Bonn, Germany; 2 Institute of Physiological Chemistry, University Medical Center of the Johannes Gutenberg University Mainz, Mainz, Germany; 3 Insitute for Clinical Chemistry and Clinical Pharmacology, University Hospital Bonn, Bonn, Germany; 4 Department of Molecular Psychiatry, University Hospital Bonn, Bonn, Germany; Max Delbruck Centrum fur Molekulare Medizin Berlin Buch, GERMANY

## Abstract

**Background:**

The endocannabinoid (eCB) 2-arachidonoylglycerol (2-AG) is a known modulator of inflammation and ligand to both, pro-inflammatory cannabinoid receptor 1 (CB1) and anti-inflammatory CB2. While the role of both receptors in atherogenesis has been studied extensively, the significance of 2-AG for atherogenesis is less well characterized.

**Methods:**

The impact of 2-AG on atherogenesis was studied in two treatment groups of ApoE^-/-^ mice. One group received the monoacylglycerol lipase (MAGL)-inhibitor JZL184 [5 mg/kg i.p.], which impairs 2-AG degradation and thus causes elevated 2-AG levels, the other group received vehicle for four weeks. Simultaneously, both groups were fed a high-cholesterol diet. The atherosclerotic plaque burden was assessed in frozen sections through the aortic sinus following oil red O staining and infiltrating macrophages were detected by immunofluorescence targeting CD68. *In vitro*, the effect of 2-AG on B6MCL macrophage migration was assessed by Boyden chamber experiments. Transcription of adhesion molecules and chemokine receptors in macrophages was assessed by qPCR.

**Results:**

As expected, application of the MAGL-inhibitor JZL184 resulted in a significant increase in 2-AG levels in vascular tissue (98.2 ± 16.1 nmol/g vs. 27.3 ± 4.5 nmol/g; n = 14–16; p < 0.001). ApoE^-/-^ mice with elevated 2-AG levels displayed a significantly increased plaque burden compared to vehicle treated controls (0.44 ± 0.03 vs. 0.31 ± 0.04; n = 14; p = 0.0117). This was accompanied by a significant increase in infiltrating macrophages within the atherosclerotic vessel wall (0.33 ± 0.02 vs. 0.27 ± 0.01; n = 13–14; p = 0.0076). While there was no alteration to the white blood counts of JZL184-treated animals, 2-AG enhanced macrophage migration *in vitro* by 1.8 ± 0.2 -fold (n = 4–6; p = 0.0393) compared to vehicle, which was completely abolished by co-administration of either CB1- or CB2-receptor-antagonists. qPCR analyses of 2-AG-stimulated macrophages showed an enhanced transcription of the chemokine CCL5 (1.59 ± 0.23 –fold; n = 5–6; p = 0.0589) and its corresponding receptors CCR1 (2.04 ± 0.46 -fold; n = 10–11; p = 0.0472) and CCR5 (2.45 ± 0.62 –fold; n = 5–6; p = 0.0554).

**Conclusion:**

Taken together, elevated 2-AG levels appear to promote atherogenesis *in vivo*. Our data suggest that 2-AG promotes macrophage migration, possibly by the CCL5-CCR5/CCR1 axis, and thereby contributes to vascular inflammation. Thus, decreasing vascular 2-AG levels might represent a promising therapeutic strategy in patients suffering from atherosclerosis and coronary heart disease.

## 1. Introduction

Atherosclerosis and its clinical manifestations coronary, peripheral and carotid artery disease are the leading cause of death worldwide and its increasing prevalence necessitates novel antiatherosclerotic strategies [1]. While the prevention and treatment of cardiovascular risk factors is still the primary therapeutic approach to atherosclerotic disease, novel antiinflammatory treatment strategies have recently been proven efficacious and safe [2]. The chronic inflammatory response that underlies atherogenesis is mediated amongst others by the innate immune system (reviewed in [3]) which expresses components of the endocannabinoid system. The endocannabinoid system is a potent regulator of inflammation in various models of inflammatory disease [4–9]. It consists of the cannabinoid receptors (CB1 and CB2), their endogenous ligands, i.e. the endocannabinoids, their synthesizing and degrading enzymes, as well as putative endocannabinoid transporters [10–12].

Amongst the endocannabinoids, N-arachidonoylethanolamide (anandamide, AEA) and 2-arachidonoylglycerol (2-AG) are the two best studied CB-receptor ligands with similar affinity to both, CB1 and CB2 receptor [13]. While most studies have attributed atherogenic and pro-infammatory properties to CB1, CB2 is considered to be anti-inflammatory and atheroprotective [5;6]. Being ligands to both, CB1 and CB2, the role of the endocannabinoids in atherogenesis is still not fully elucidated. So far, only few studies have addressed the role of 2-AG in atherosclerosis: Montecucco et al. have paved the way demonstrating that 2-AG is elevated in aortic tissue of ApoE^-/-^ mice following a high cholesterol diet. Furthermore, they demonstrated that 2-AG increases human monocyte migration which potentially could increase vascular inflammation [14]. In previous work by our group, we investigated the consequences of a myeloid-specific knockout of 2-AG-synthesizing enzyme diacylglycerol lipase α (DALGα) in ApoE^-/-^ mice. Using a chimeric mouse model, we were able to demonstrate that the impairment of myeloid 2-AG synthesis decreases vascular inflammation and atherosclerotic lesion formation [15]. Interestingly, one study has already examined the consequences of elevated 2-AG levels on atherosclerosis in a genetic knockout mouse model [16]. While the authors of that study found 2-AG to enhance atherogenesis, they also reported a reduced relative lipid and macrophage plaque content, with an increase in collagen content and fibrous cap thickness. This led the authors to conclude that 2-AG might be beneficial in the treatment of atherosclerosis since it might stabilize atherosclerotic plaques even though it augments atherosclerotic plaque size. In the present study, we sought to contribute to the ongoing discussion about the impact of elevated 2-AG levels on atherogenesis *in vivo*. We therefore treated ApoE^-/-^ mice with JZL184, a selective inhibitor of the 2-AG-degrading enzyme monoacylglycerol lipase (MAGL) or vehicle control respectively. Furthermore, we studied the influence of 2-AG on murine macrophage migration and on the expression of adhesion molecules and chemokine receptors *in vitro*. Here we report 2-AG to increase the atherosclerotic plaque burden and suggest a proinflammatory role of 2-AG in atherogenesis.

## 2. Methods

Animals were kept at 22°C room temperature and a 12 hour dark-light-cycle. Access to drinking water and rodent chow was unrestricted. Laboratory animal use and all experimental procedures were in accordance with institutional guidelines and German animal protection law. Approval was granted by the responsible german institute, the North Rhine Westphalian State Agency for Nature, Environment and Consumer Protection (reference number 84–02.04.2014.A419) which considered ethics and welfare aspects of all animal protocols in their approval.

### 2.1 Atherogenic diet and treatment with JZL184 of ApoE^-/-^ mice

ApoE^-/-^ mice (C57BL/6J genetic background; Charles River, Wilmington, USA) were treated with intraperitoneal injections of either the selective MAGL inhibitor JZL 184 (5 mg/kg body weight) or same concentrations of vehicle (DMSO) three times per week for a total of four weeks. Initially, a dose-response experiment was conducted by treating ApoE^-/-^ mice with increasing concentrations of JZL184 (0.1 mg/kg, 0.5 mg/kg, 5 mg/kg). ApoE^-/-^ mice (n = 3 mice per dosage) were injected i.p. three times for one week. Plasma and aortic tissue were collected 24 hours after the third injection in order to insure a persistent elevation of 2-AG (S1 Fig). 5 mg/kg yielded the highest plasma and tissue levels of 2-AG. Higher concentrations than 5 mg/kg were avoided in order not to lose specificity for MAGL inhibition over e.g. FAAH inhibition and to limit potential neurological side effects.

Both solutions, JZL184 and DMSO control, were diluted in sterile PBS prior to injection. The final concentration of DMSO was 1% (v/v) in either solution. 5% (v/v) of Kolliphor (Sigma Aldrich) is essential to emulsify JZL 184 and was added to both solutions in equal amounts. Final concentrations thus yielded 1% (v/v) of DMSO and 5% (v/v) of Kolliphor in the vehicle solution. During the treatment period, mice were fed a high-fat and high-cholesterol diet containing 21% (w/w) fat, 19.5% (w/w) casein, and 1.25% (w/w) cholesterol (Ssniff, Soest, Germany).

Upon completion of the treatment period mice were sacrificed by cervical dislocation under 5% (v/v) isoflurane anaesthesia (Abbott Laboratories, Chicago, USA). The final injection of JZL184 or vehicle was 4 hours prior to sacrifice.

### 2.2. Extraction of endocannabinoids and LC-MRM quantification

Liquid chromatography-multiple reaction monitoring (LC-MRM) was used to quantify concentrations of anandamide (AEA), 2-arachidonoylglycerol (2-AG), and arachidonic acid (AA) in murine plasma and aortic tissue as described [17]. Frozen aortic tissue samples were transferred in pre-cooled extraction tubes containing cold steel balls. The samples were then spiked with 50 μl solution of deuterated eCBs in acetonitrile, serving as internal standards. 250 μl 0.1 M formic acid was added as homogenization buffer and 300 μl ethylacetate/n-hexane (9:1, v/v) as extraction solvent. The samples were homogenized for 40 seconds at 30 Hz using a tissue lyser. (Qiagen GmbH, Hilden, Germany). The homogenates were then centrifuged (15 minutes; 16,000 x g; 4 °C) and kept at -20 °C for 20 minutes allowing the aqueous phase to freeze. The organic phase was recovered in 96 well plates, evaporated under a gentle stream of N_2_ and reconstituted in 50 μl acetonitrile/H_2_O (1:1, v/v) using an automated pipetting machine. The samples were then subjected to LC-MRM analysis. The protein content was determined in the aqueous phase using a BCA assay. For plasma eCBs extraction, 100 μl plasma per sample were first thawed at 4°C, and then 50 μl spike solution of internal standards and 250 μL ethyl acetate/n-hexane (9:1, v/v) were added to the samples. The samples were vortexed for 30 seconds and then centrifuged (10 minutes; 16,000 x g; 4°C). The samples were then kept at -20°C for 10 min, the upper organic phase was recovered in 96 well plates, evaporated to dryness and the extract reconstituted in 50 μl acetonitrile/H_2_O (1:1, v/v) for LC-MRM analysis. Sacrifice, tissue sampling and endocannabinoid extraction methods were highly standardized, in order to minimize differences between *in vivo* endocannabinoid levels and post mortem endocannabinoid levels due to potential post mortem synthesis or degradation of 2-AG [18]. Throughout the eCB extraction procedure, samples were kept on ice to prevent artificial alteration to the endogenous levels of the eCBs. The eCB values were normalized to the aortic protein content, or to the plasma volume respectively.

### 2.3. Assessment of body weight, plasma cholesterol, blood pressure, heart rate

Body weight was measured on days 0 and 28 of the feeding period using a Kern EW 3000-2M balance (Kern & Sohn GmbH, Balingen, Germany). Plasma cholesterol levels were measured by gas chromatography, followed by flame ionisation detection, as described earlier [19].

Blood pressure and heart rate were measured by a non-invasive Volume Pressure Recording system (Kent Scientific Corporation, Torrington, USA). Mice were accustomed to the recording system prior to data collection. Actual recording of the measurements was accomplished on the three consecutive days following the acclimatization period.

### 2.4. Histological characterization of atherosclerotic plaques

Hearts and the proximal part of the ascending aorta were embedded upright in tissue freezing medium and were snap frozen at -80 °C. The aortic sinus was sliced using a Leica CM 1900 cryostat (Leica Biosystems GmbH, Wetzlar, Germany) at a thickness of 8 μm per section. 10 consecutive slices were placed on 10 individual object slides at position 1. Slices 11–20 were placed on these same 10 individual object slides at position 2 and so on. This resulted in a total of 10 object slides on which neighboring sections have initially been 80 μm apart. Sections displaying all three cusps of the aortic valve have been considered for plaque analysis. Sections above or below the aortic valve on which not all three cusps could be identified were not included in the analysis. On average, 4 sections per heart were analyzed.

Then, atherosclerotic plaques were stained using oil red O (Sigma-Aldrich, St. Louis, USA) as described [15]. In brief, sections were dried, fixated with 4% (w/v) paraformaldehyde (PFA) in PBS for 45 minutes and dehydrated with 60% (v/v) isopropanol for 5 minutes. Hereafter slides were stained in oil red O solution for 15 minutes and costained with haematoxylin for 30 seconds.

Macrophages within the atherosclerotic vessel wall were detected by immunofluorescence targeting CD68 (primary antibody: α-CD68 rat IgG2a, Acris antibodies GmbH, Herford, Germany; secondary antibody: Cy3 AffiniPure Donkey anti-Rat IgG, Jackson ImmunoResearch Laboratories, Inc.). Briefly, slides were fixed in acetone for 20 minutes, washed in PBS and blocked in 1% (w/v) BSA in PBS for 30 minutes. Afterwards, sections were incubated with the primary antibody (1:100) at 4 °C overnight. Hereafter, slides were washed with PBS and the secondary antibody (1:500) was administered at room temperature in the dark for 1 hour. Nuclei were stained using Vectashield mounting medium with DAPI (Vector Laboratories, Burlingame, USA).

Smooth muscle cells were stained using an immunofluorescent antibody targeting alpha smooth muscle actin (α-SMA; Anti-Actin, α-Smooth Muscle—Cy3 antibody, Sigma-Aldrich). Slides were dried and dehydrated in 100% (v/v) acetone for 20 minutes. Hereafter, slides were rinsed with PBS, blocked with 2% (w/v) BSA in PBS, and then incubated with the Cy3-conjugated α-SMA-antibody at room temperature for 1 hour. Finally, nuclei were stained using Vectashield mounting medium with DAPI (Vector Laboratories).

Collagen content was visualized by Picro Sirius staining (Direct Red 80, Sigma-Aldrich). Slides were fixed in an ascending series of 70% (v/v), 90% (v/v), 100% (v/v) ethanol for 10 minutes at each concentration. Then slides were rinsed in deionised water. Hereafter, slides were stained with haematoxylin (Carl Roth GmbH + Co. KG, Karlsruhe, Germany) for 30 seconds and with Direct Red for 15 minutes. Then, slides were fixed in a descending series of 100% (v/v), 90% (v/v), 70% (v/v) ethanol for 10 minutes at each concentration. Finally, slides were covered with Entellan^®^ (Merck, Darmstadt, Germany).

Microscopic pictures were obtained with a Zeiss Axiovert 200M microscope (Carl Zeiss Jena GmbH, Jena, Germany) using Axiovision 4.8 software (Carl Zeiss Jena GmbH). Plaque sizes and positively stained areas were quantified using the feature “measure—outline” of Axiovision 4.8 software. Areas were calculated by the software in μm^2^. Subsequently, these areas were divided by the area of the vessel wall (difference between the area within the outer circumference of the aorta and the area of the aortic lumen) in order to correct for potential differences in the diameter of the aortic roots which may have resulted from sectioning artifacts (e.g. stretching or compression of the slices).

### 2.5. Flow cytometry of circulating immune cells

Following lysis of erythrocytes, leukocytes were stained for CD11b, CD3, CD19, Ly6C, and Ly6G (clones M1/17, 17A2, 1D3, RB6-8C5, AL-21, BD Biosciences, San Jose, USA). Prevalence of these surface markers within a pre-specified leukocyte gate was determined after measuring 50,000 counts. Flow cytometry plots showing gating strategies are shown in S2 Fig. Unstained samples and isotype identical antibodies were used as negative controls. Data analysis was performed using FlowJo software (FlowJo LLC, Ashland, USA). Characteristics of all antibodies used for histological and flow cytometry staining are detailed in S1 Table.

### 2.6. Macrophage migration and proliferation

B6MCL macrophages were generously provided by Professor Eicke Latz (Bonn, Germany) [20]. B6MCL is an immortalized murine cell line derived from wildtype C57BL6 mice using a J2 recombinant retrovirus. Migration of B6MCL macrophages was assessed by modified Boyden chamber experiments in a 24-well-plate format. Upper and lower chamber were separated by an 8.0 μm pore polycarbonate membrane insert (Corning, New York, USA). 350,000 cells were seeded into the upper chamber, containing hunger medium (DMEM/F12, Thermo Fisher Scientific Inc., Waltham, USA) without FBS. The lower chamber contained DMEM/F12 with 10% (v/v) FBS. 1 μM 2-AG was added to the lower chamber to enhance macrophage migration, its solvent DMSO served as negative control. In order to test for CB-receptor specificity of 2-AG effects, the selective CB1-inhibitor AM281 [1 μM] and the selective CB2-inhibitor AM630 [1 μM] were added to the upper chamber. Cells were allowed to migrate for 90 minutes. Hereafter, the inserts were washed with PBS, fixated with 2% (w/v) PFA and stained with Vectashield mounting medium with DAPI (Vector Laboratories). Microscopic pictures of the lower side of the polycarbonate membrane were obtained with a Zeiss Axiovert 200M microscope (Carl Zeiss Jena GmbH) and counted automatically using Image J 1.48v software (National Institute of Health, Bethesda, USA).

In an additional experimental approach, B6MCL macrophages were preconditioned with 2-AG [1 μM] or DMSO respectively prior to the migration experiment. B6MCL were kept in hunger medium supplemented with 2-AG [1 μM] or 0.1‰ (v/v) DMSO respectively for 15 minutes before being seeded into the upper chamber (350,000 cells per well). The lower chamber contained DMEM/F12 with 10% (v/v) FBS or DMEM/F12 with 10% (v/v) FBS supplemented with CCL5 [1 μg/ml]. The lower chamber did not contain 2-AG in this experiment.

The influence of 2-AG on macrophage proliferation was studied using the FITC bromodeoxyuridine (BrdU) Flow Kit by BD Biosciences according to the manufacturer’s instructions. In brief, B6MCL macrophages were seeded in 6-well plates at a density of 320,000 cells per well and allowed to adhere overnight. Then 2-AG was added to the wells at a final concentration of 1 μM for 1 hour. Negative control wells were treated with equal amounts of the solvent DMSO. Following stimulation, macrophages were pulse-labeled with 10 μM BrdU for 45 minutes allowing for analysis of cell cycle distribution. Hereafter, cells were fixed, permeabilized and stained with a FITC-conjugated BrdU antibody. Total DNA content was stained with 20 μl of 7-AAD per well. Finally, 100,000 cells per well were counted using a FACSCalibur flow cytometer (BD Biosciences) and data analysis was performed using FlowJo software (FlowJo LLC).

### 2.7. Macrophage stimulation and qPCR analyses

B6MCL macrophages were grown in Dulbecco’s modified eagle medium (Thermo Fisher Scientific Inc.) supplemented with 10% (v/v) FBS and 1% (v/v) penicillin and streptomycin at 37°C and 5% CO_2_. At 80% confluence, cells were stimulated with 2-AG [1 μM] or DMSO [0.1‰ (v/v)] respectively for four hours. Hereafter, cells were rinsed in PBS and harvested using 1 ml Trizol^®^ (Ambion life technologies / Thermo Fisher Scientific Inc.). By addition of chloroform (Merck, Darmstadt, Germany) and following centrifugation (18,000 x g and 4°C, 15 minutes), phases were separated with the upper phase being the RNA-containing phase. The RNA-containing phase was then transferred into another reaction tube and RNA was precipitated by addition of isopropanol. RNA was washed twice with ethanol, dried and resuspended in RNAse-free water. 2 μg of RNA were reversely transcribed (Omniscript RT kit; Qiagen GmbH). cDNA was used to perform qPCR analyses using TaqMan^®^ probes (Thermo Fisher Scientific Inc.) and the appropriate master mix. CDNA amplification was then performed using a 7500 Fast Real-Time PCR system and 7500 software v.2.0.6 (both Thermo Fisher Scientific Inc.). Details about the TaqMan^®^ probes used in this study are listed in S2 Table.

### 2.8. Statistical analyses

Data are presented as mean ± SEM. Statistical differences of continuous variables were determined using Microsoft excel software (Microsoft, Redmond, USA) and Origin 8.0 software (OriginLab Corporation, Northampton, USA) For comparison of two groups, unpaired Student’s two-sided t-tests was applied. For the comparison of 3 or more groups, one-way ANOVA and subsequent Bonferroni correction was performed. P < 0.05 was considered statistically significant.

## 3. Results

### 3.1. Pharmacological inhibition of MAGL with JZL184 increases both plasma and vascular tissue 2-AG levels in ApoE^-/-^ mice

ApoE^-/-^ mice were treated with the MAGL-inhibitor JZL184 or vehicle for 4 weeks, while being fed a high fat and high cholesterol diet. As expected, ApoE^-/-^ mice, treated with the selective MAGL inhibitor JZL184 displayed significantly increased 2-AG levels compared to vehicle-treated controls. The mean plasma 2-AG level in the JZL184-treated group was 40.4 ± 6.2 pmol/ml compared to 26.2 ± 2.3 pmol/ml in the vehicle treated control group (n = 13; p = 0.0433). Meanwhile, plasma levels of both, arachidonic acid (AA) and the endocannabinoid AEA, were unaffected by JZL184 treatment (AA: 6.1 ± 0.6 nmol/ml vs. 5.9 ± 0.5 nmol/ml; n = 14–16; p > 0.05; AEA: 1.3 ± 0.1 pmol/ml vs. 1.1 ± 0.1 pmol/ml; n = 14–16; p > 0.05).

The elevation of 2-AG induced by JZL184 was even more pronounced within the aortic tissue yielding 98.2 ± 16.1 nmol/g compared to 27.3 ± 4.5 nmol/g in the vehicle treated control group (n = 14–16; p = 0.0004). Again, there was no difference in the aortic tissue levels of AA and AEA between the two treatment groups (AA: 1,781 ± 214 nmol/g vs. 2,006 ± 338 nmol/g; n = 14–16; p > 0.05; AEA: 161 ± 27 pmol/g vs. 160 ± 26 pmol/ml; n = 14–16; p > 0.05).

### 3.2. Body weight, plasma cholesterol, blood pressure, heart rate

The clinical parameters body weight, blood pressure and heart rate within the two groups are detailed in Table 1. None of these parameters was significantly different between the two groups. Plasma cholesterol levels and phytosterols are listed in Table 2—again, no differences were measured between the two groups.

**Table 1:** Clinical parameters. Clinical parameters were assessed at the end of the feeding period. Body weight, arterial blood pressure and heart rate were similar in both groups. Data are presented as mean ± standard error of the mean; n = 14–16; p-values as stated, assessed by student’s t-test. DMSO, dimethyl sulfoxide; JZL184, inhibitor of monoacylglycerol lipase.

	DMSO	JZL184	p
Body weight [g]	24.9 ± 1.1	22.8 ± 1.1	0.1950
Systolic blood pressure [mmHg]	124.2 ± 5.6	136.8 ± 5.8	0.1434
Diastolic blood pressure [mmHg]	95.6 ± 5.7	91.7 ± 5.5	0.6372
Heart rate [bpm]	704 ± 15	692 ± 24	0.7016

**Table 2:** Assessment of cholesterol and phytosterols. Cholesterol and phytosterol plasma levels were assessed at the end of the feeding period. There were no differences between the two treatment groups. Data are presented as mean ± standard error of the mean; n = 9–10; p-values as stated, assessed by student’s t-test. DMSO, dimethyl sulfoxide; JZL184, inhibitor of monoacylglycerol lipase.

	DMSO	JZL184	p
Cholesterol [mg/dl]	1291 ± 67	1234 ± 38	0.8465
Cholestanol [mg/dl]	2.66 ± 0.12	2.91 ± 0.13	0.1783
Lathosterol [mg/dl]	0.62 ± 0.05	0.59 ± 0.04	0.6541
Campesterol [mg/dl]	0.87 ± 0.05	0.93 ±0.04	0.3492
Sitosterol [mg/dl]	0.43 ± 0.03	0.49 ± 0.03	0.1442

### 3.3. JZL184-treatment increases atherogenesis in ApoE^-/-^ mice

**Fig 1:**
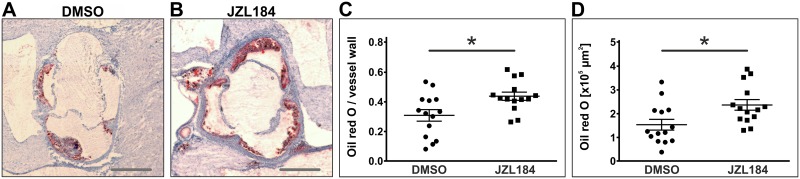
Assessment of atherosclerotic plaque size by oil red O staining. Frozen sections through the aortic sinus of both DMSO-treated control mice and JZL184-treated mice with elevated 2-AG levels were stained using oil red O to visualize atherosclerotic plaques (A, B). Cumulative plaque area was divided by the area of the vessel wall to obtain the relative plaque burden (C). Cumulative plaque areas are depicted in panel D. Data are presented as mean ± standard error of the mean; n = 14; *, p < 0.05, assessed by student’s t-test. Scale bar, 500 μm. DMSO, dimethyl sulfoxide; JZL184, inhibitor of monoacylglycerol lipase.

Atherosclerotic plaques were visualized by oil red O staining of frozen sections through the aortic sinus and oil red O-positive areas were normalized to the size of the vessel wall. ApoE^-/-^ mice that had been treated with JZL184 showed a significantly increased atherosclerotic plaque burden compared to vehicle-treated controls yielding 0.44 ± 0.03 vs. 0.31 ± 0.04 (n = 14; p = 0.0117; Fig 1A–1C).

**Fig 2:**
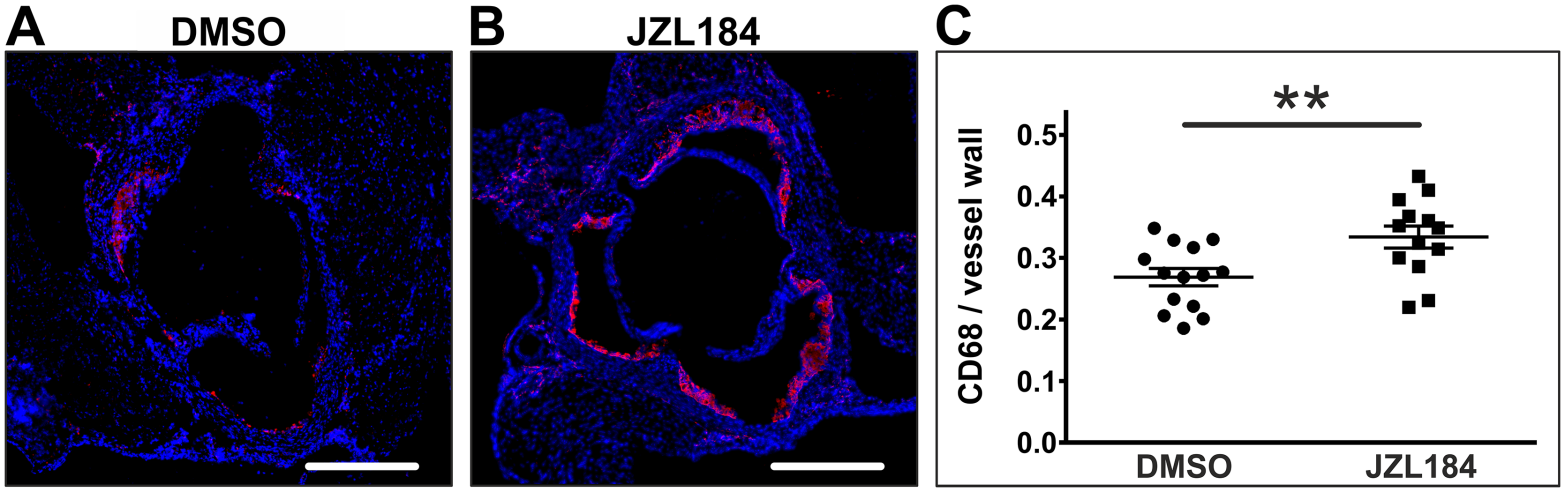
Quantification of CD68-positive macrophages and monocytes. Frozen sections through the aortic sinus of both DMSO-treated control mice and JZL184-treated mice with elevated 2-AG levels were stained for CD68 to visualize macrophages and monocytes (A, B). Positively stained areas were divided by the area of the vessel wall to obtain relative vessel infiltration (C). Data are presented as mean ± standard error of the mean; n = 13–14; **, p < 0.01, assessed by student’s t-test. Scale bar, 500 μm. DMSO, dimethyl sulfoxide; JZL184, inhibitor of monoacylglycerol lipase.

Infiltrating macrophages within the atherosclerotic vessel wall were identified following immunofluorescence staining of CD68. Macrophage infiltration into the atherosclerotic vessel wall was significantly increased in JZL184-treated mice compared to DMSO-treated controls (0.33 ± 0.02 vs. 0.27 ± 0.01; n = 13–14; p = 0.0076; Fig 2A–2C).

**Fig 3:**
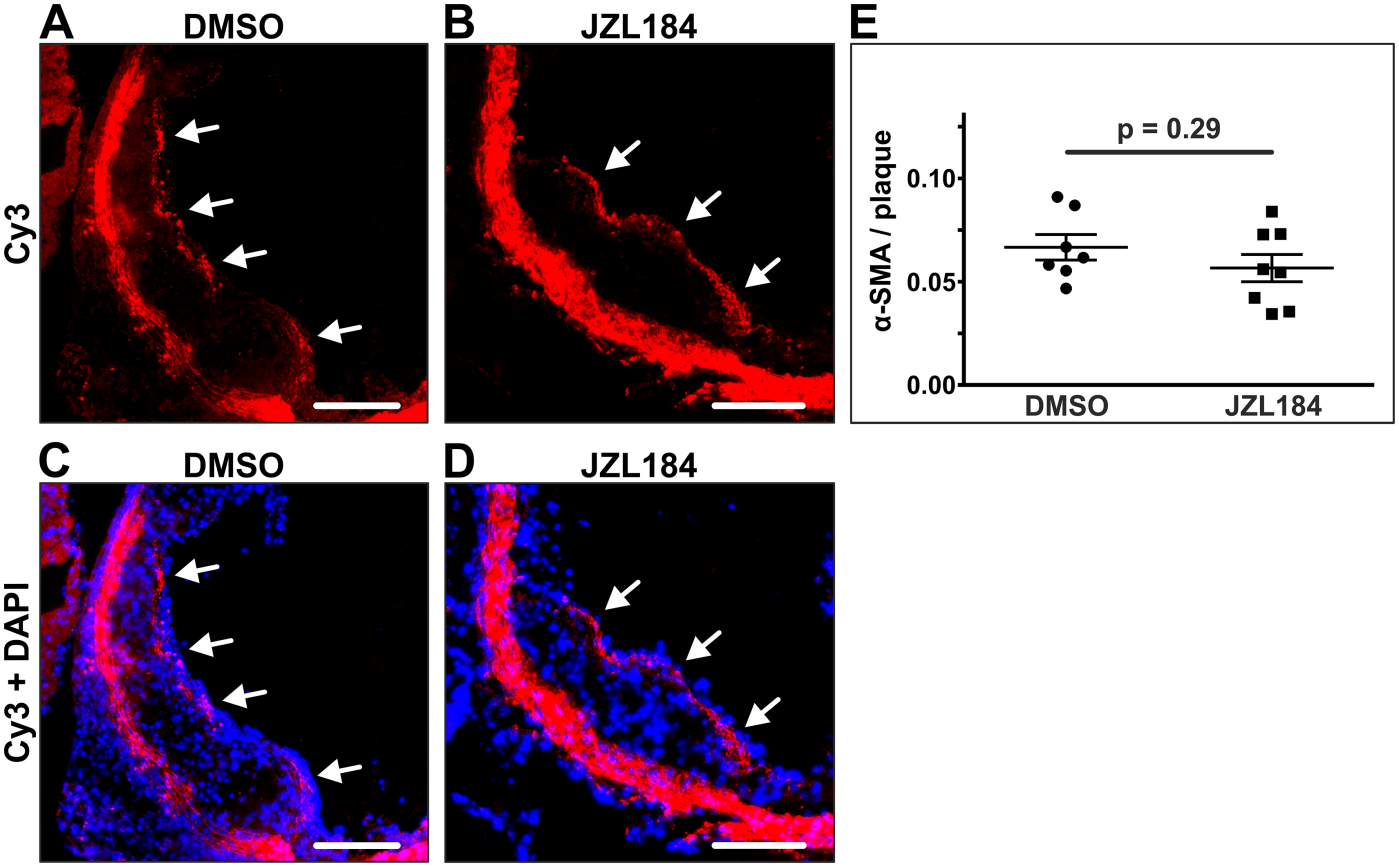
Quantification of smooth muscle cells within atherosclerotic plaques. Smooth muscle cells were stained in the aortic sinus using an immunofluorescent antibody targeting alpha smooth muscle actin. Significant amounts of smooth muscle cells were detected in 7 DMSO-treated animals and in 8 JZL184-treated animals (A, B; white arrows). The proportion of smooth muscle cells to the plaque area was unaffected by JZL184 (C). Data are presented as mean ± standard error of the mean; n = 7–8; p-value as indicated, assessed by student’s t-test. Scale bar, 200 μm. α-SMA, alpha smooth muscle actin; Cy3, cyanine 3; DAPI, 4',6-diamidino-2-phenylindole; DMSO, dimethyl sulfoxide; JZL184, inhibitor of monoacylglycerol lipase.

In order to further characterize plaque morphology, smooth muscle cells were stained in frozen sections through the aortic sinus. Treatment with JZL184 showed no effect on the smooth muscle cell content of atherosclerotic plaques. α-SMA staining yielded quantifiable amounts of smooth muscle cells in 7 out of 14 animals in the DMSO group and in 8 out of 14 animals of the JZL184 group with a similar proportion to the plaque area in both groups (0.067 ± 0.006 vs. 0.057 ± 0.007; n = 7–8; p = 0.2903; Fig 3A–3C).

**Fig 4:**
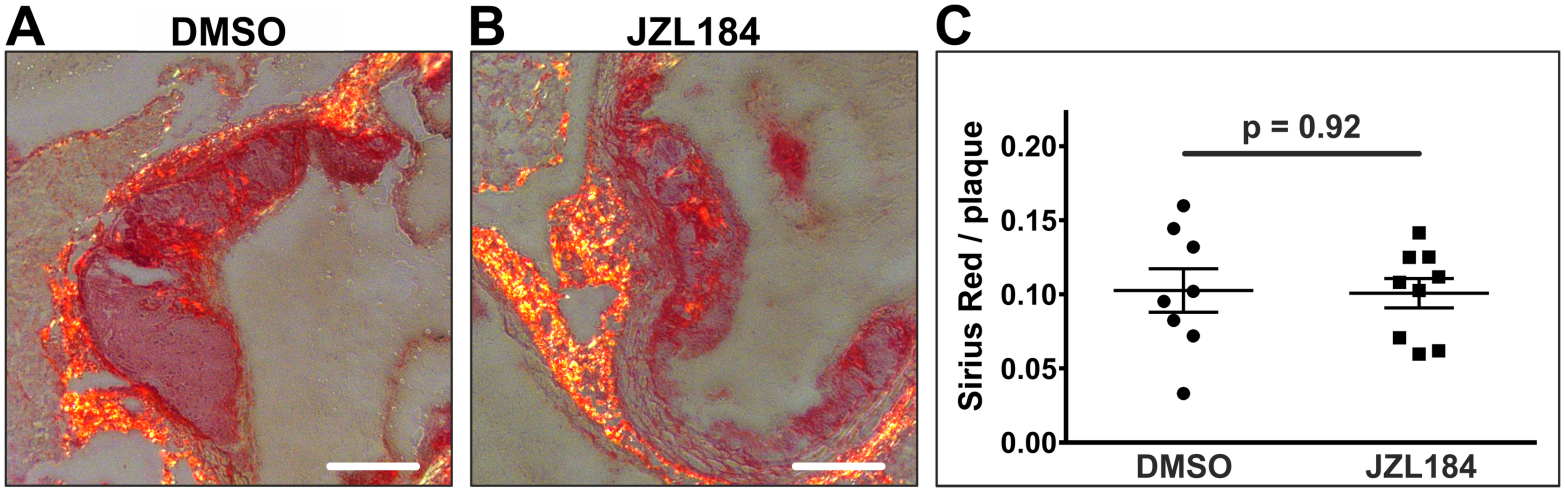
Staining of collagen fibers. Collagen fibers were visualized in the aortic sinus by Picro Sirius staining. Significant amounts of collagen fibers were detected in 8 DMSO-treated animals and in 9 JZL184-treated animals (A, B). Quantity and distribution pattern of collagen fibers did not differ between the two groups (C). Data are presented as mean ± standard error of the mean; n = 8–9; p-value as indicated, assessed by student’s t-test. Scale bar, 200 μm. DMSO, dimethyl sulfoxide; JZL184, inhibitor of monoacylglycerol lipase.

Similarly, collagen fibers did not differ between the two groups, neither in terms of quantity nor in terms of the distribution pattern (0.10 ± 0.01 vs. 0.10 ± 0.01; n = 8–9; p = 0.9158; Fig 4A–4C).

Finally, necrotic cores were identified on haematoxylin- and DAPI-stained sections. Necrotic cores appeared in 11 animals of either group and yielded comparable sizes (0.09 ± 0.02 vs. 0.09 ± 0.01; n = 11; p = 0.7647).

### 3.4 Cell counts of circulating leukocytes and bone marrow cells were unaffected by JZL184-treatment

Macrophage accumulation within the atherosclerotic vessel wall was significantly increased in mice treated with JZL184 compared to vehicle-treated mice, which might have been caused by an enhanced leukopoiesis or an increase in circulating leukocytes. In order to rule out theses systemic effects of JZL184, we examined leukocyte subsets in bone marrow and peripheral blood samples using flow cytometry. Leukocyte subsets in both, bone marrow and peripheral blood were unaffected by JZL184-treatment (Table 3).

**Table 3:** Flow cytometry-based analysis of bone marrow and peripheral blood leukocytes. Leukocytes were stained for CD11b, CD3, CD19, Ly6C, and Ly6G. Prevalence of these surface markers within a pre-specified leukocyte gate was determined after measuring 50,000 counts. Data are presented as mean ± standard error of the mean; n = 11–13; p-values as stated, assessed by student’s t-test. CD, cluster of differentiation; DMSO, dimethyl sulfoxide; JZL184, inhibitor of monoacylglycerol lipase; Ly6C, lymphocyte antigen 6C; Ly6G, lymphocyte antigen 6G.

A—bone marrow			
Antigen	DMSO	JZL184	p
CD19 (B-cells) [x10^6^ cells per femur]	28.06 ± 5.50	25.47 ± 6.06	0.7549
CD11b^+^Ly6C^+^Ly6G^+^ (neutrophils) [x10^6^ cells per femur]	106.49 ± 13.17	84.76 ± 18.91	0.3581
CD11b^+^Ly6C^low^Ly6G^-^ (patrolling monocytes) [x10^6^ cells per femur]	36.96 ± 6.93	36.33± 5.73	0.9451
CD11b^+^Ly6C^high^Ly6G^-^ (inflammatory monocytes) [x10^6^ cells per femur]	15.27 ± 2.10	12.43 ± 3.08	0.4574
B—peripheral blood			
CD19 (B-cells) [per μl blood]	2,897 ± 611	3,994 ± 898	0.4442
CD3 (T-cells) [per μl blood]	686 ± 133	929 ± 211	0.4667
CD11b^+^Ly6C^+^Ly6G^+^ (neutrophils) [per μl blood]	1,814 ± 423	2,892 ± 666	0.2175
CD11b^+^Ly6C^low^Ly6G^-^ (patrolling monocytes) [per μl blood]	2,926 ± 449	3,894 ± 714	0.2182
CD11b^+^Ly6C^high^Ly6G^-^ (inflammatory monocytes) [per μl blood]	389 ± 68	552 ± 99	0.2182

### 3.5. 2-AG enhances macrophage migration *in vitro*

**Fig 5:**
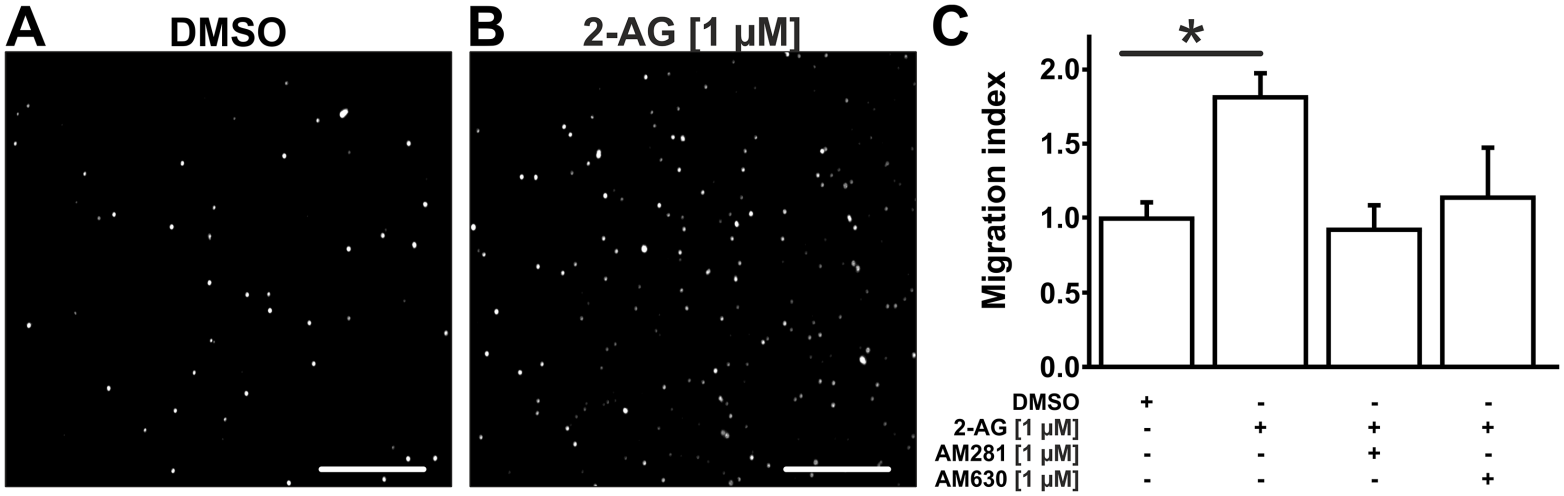
2-AG promotes B6MCL macrophage migration as assessed by modified Boyden chamber experiments. B6MCL macrophage migration was assessed by modified Boyden chamber experiments. 350,000 cells were seeded per well and allowed to migrate for 90 minutes. Cells adherent to the lower side of the polycarbonate membrane were counted automatically using Image J 1.48v software following DAPI staining. Addition of 2-AG [1 μM] to the lower chamber resulted in an increased migration of B6MCL macrophages (B) compared to DMSO (A). This effect was blunted by addition of CB1-inhibitor AM281 [1 μM] and by CB2-inhibitor AM630 [1 μM] (C). Data are presented as mean ± standard error of the mean; n = 4–6; *, p < 0.05, assessed by ANOVA and Bonferroni correction. Scale bar, 300 μm. 2-AG, 2-arachidonoylglycerol; AM281, selective inhibitor of CB1; AM630, selective inhibitor of CB2; DMSO, dimethyl sulfoxide.

Since elevated 2-AG levels did not influence circulating leukocyte counts, we suspected 2-AG to locally enhance macrophage migration or proliferation at the site of vascular inflammation. Hence, migration of B6MCL macrophages was assessed by modified Boyden chamber experiments. Indeed, 2-AG [1 μM] led to a significant increase in macrophage migration by 1.8 ± 0.2 -fold (n = 4–6; p = 0.0393) compared to vehicle when added to the lower chamber (Fig 5A–5C). This increase in macrophage migration was prevented by addition of the selective CB1-inhibitor AM281 [1 μM] to the upper chamber (0.9 ± 0.2 -fold; n = 3; p_vs. control_ > 0.05). Addition of the selective CB2-inhibitor AM630 similarly decreased 2-AG induced macrophage migration (1.1 ± 0.3 -fold; n = 3; p_vs. control_ > 0.05; Fig 5C).

Interestingly, B6MCL migration was also enhanced when 2-AG was not used as a chemoattractant (lower chamber), but when macrophages were preconditioned with 2-AG prior to the migration experiment. Preconditioning of B6MCL with 2-AG [1 μM] significantly enhanced migration towards DMEM/F12 with 10% (v/v) FBS compared to preconditioning with DMSO (3.8 ± 0.2 -fold versus DMSO; n = 4; p < 0.0001). Addition of CCL5 to the lower chamber did not synergistically enhance migration of 2-AG preconditioned macrophages (2.2 ± 0.3 -fold versus DMSO; n = 4; p_vs. DMSO_ = 0.0073; p_vs. 2-AG without CCL5_ = 0.2010).

**Fig 6:**
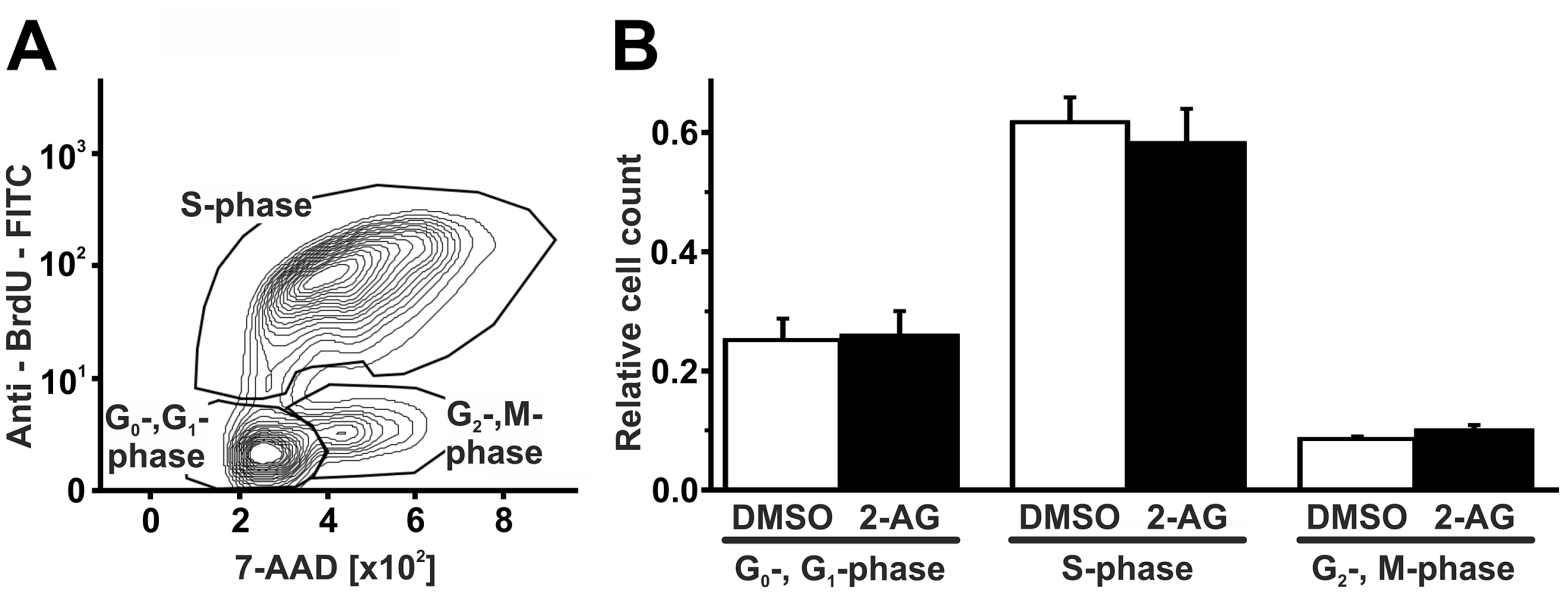
2-AG does not influence proliferation of B6MCL macrophages. B6MCL macrophage proliferation was assessed using a flow cytometry-based BrdU assay. There were no changes in cell cycle distribution following treatment with 2-AG [1μM] for 60 minutes. Data are presented as mean ± standard error of the mean; n = 3; p > 0.05, assessed by ANOVA and Bonferroni correction. 2-AG, 2-arachidonoylglycerol; 7-AAD, 7-Aminoactinomycin D; BrdU, bromodeoxyuridine; DMSO, dimethyl sulfoxide.

Hereafter, the influence of 2-AG on macrophage proliferation was assessed using a flow-cytometry-based BrdU assay. 2-AG [1 μM] did not influence cell cycle distribution of B6MCL macrophages compared to DMSO-treated controls. Among 2-AG treated cells, 25.9 ± 4.1% were in G0-/G1-phase, 10.0 ± 0.8% were in G2-/M-phase, and 58.3 ± 5.1% were in S-phase. Cell cycle distribution of DMSO-treated cells yielded 25.2 ± 3.6% for G0-/G1-phase, 8.6 ± 0.4% for G2-/M-phase, and 61.8 ± 4.1% for S-phase (n = 3; p > 0.05 for either phase; Fig 6A and 6B).

### 3.6. 2-AG enhances transcription of adhesion molecules and chemokine receptors in macrophages and influences their cholesterol metabolism

B6MCL macrophages were stimulated with 2-AG [1 μM] for four hours. mRNA was isolated from lysed cells and qPCR analyses were performed. 2-AG-treated macrophages displayed a significant increase in CCR-1 (2.04 ± 0.46 -fold; n = 10–11; p = 0.0472) and ICAM-1 (2.09 ± 0.42 –fold; n = 5–6; p = 0.0447) mRNA levels. Furthermore, there was a marked yet not statistically significant increase in CCL5 (1.59 ± 0.23 –fold; n = 5–6; p = 0.0589), CCR5 (2.45 ± 0.62 –fold; n = 5–6; p = 0.0554), CCL2 (1.7 ± 0.40 –fold; n = 5–6; p = 0.1371) and VCAM-1 (4.59 ± 1.88 –fold; n = 5–6; p = 0.1272) mRNA levels.

Finally, we measured the influence of 2-AG on scavenger receptor CD36 which mediates cholesterol uptake and the influence of 2-AG on ABCA1 and ABCG1 which mediate cholesterol efflux. While 2-AG did not impact on ABCG1 mRNA levels (0.96 ± 0.36 –fold; n = 3; p = 0.9321), we saw a non-significant decrease in ABCA1 mRNA levels (0.57 ± 0.24 –fold; n = 3; p = 0.2518) and a significant increase in CD36 mRNA levels (8.02 ± 1.89 –fold; n = 3; p = 0.0279).

## 4. Discussion

Endocannabinoids play a crucial role in the regulation of inflammatory processes and have been linked to vascular inflammation and atherogenesis [5–7]. The present study underscores the proinflammatory and atherogenic effects of 2-AG which is the most abundantly expressed endocannabinoid.

Atherosclerosis-prone ApoE^-/-^ mice were treated with intraperitoneal injections of MAGL-inhibitor JZL184 in order to investigate the impact of raised 2-AG levels on atherogenesis *in vivo*. Pharmacological MAGL-inhibition led to a significant increase in 2-AG levels in murine plasma and aortic tissue. However, MAGL-induced hydrolysis is not only relevant to the disintegration of 2-AG. Yet, hydrolysis of 2-AG by MAGL is also an important step to the synthesis of arachidonic acid and its derivatives which partly exert proinflammatory effects themselves. It was therefore important to perform LC/MRM analyses to demonstrate that pharmacological inhibition of MAGL did not cause a reduction in AA levels. This finding seems at odds with earlier studies reporting markedly decreased AA levels in the brain following MAGL inhibition [21]. This may be explained by the fact, that hydrolysis of 2-AG by MAGL is not the main synthesizing pathway of AA in peripheral tissues. Outside the central nervous system, AA is mostly synthesized by phospholipase A2 (PLA2) enzymes via hydrolysis of phospholipids, whereas in the central nervous system hydrolysis of 2-AG by MAGL is the main synthesizing pathway of AA [22]. The latter is further corroborated by the fact that genetic disruption of PLA2 does not alter cerebral AA levels whereas peripheral AA levels are significantly diminished [23]. It is thus conceivable, that inhibition of MAGL by JZL184 affects peripheral AA levels only slightly if at all since the majority of peripheral AA is not synthesized by MAGL but by PLA2.

Likewise, MAGL inhibition did not influence endocannabinoid levels other than 2-AG. Importantly, JZL184 led to a marked increase in 2-AG levels within the aortic tissue arguing for a reasonable bioavailability of the drug at the site of inflammation within the arterial vessel wall. This is of particular relevance given that 2-AG is a locally acting transmitter that exerts its function mainly via autocrine and paracrine signaling (reviewed by [24]). Even though to our knowledge, there are currently no studies that specifically address the question which cell types contribute to vascular endocannabinoid synthesis, we can speculate that mostly the immune cells express the synthesizing and degrading enzymes for 2-AG. Based on our present work, off target effects of JZL184 cannot be ruled out. Lipid mediators other than 2-AG that are metabolized by MAGL might contribute to the observed effects. However, in the context of the available literature it is likely that 2-AG acts as a proinflammatory and atherogenic agent [15;16]. The present study did not use cannabinoid receptor deficient mice to prove the CB receptor dependence of the observed effects *in vivo*. Further studies are warranted to overcome this weakness.

Endocannabinoids are known to upregulate eating motivation via the CB1 receptor at the nucleus accumbens and hypothalamic nuclei (reviewed by [25]). Since JZL184 passes the blood-brain barrier and strongly increases cerebral 2-AG levels [26], one might suspect changes in nutritional behavior following JZL184 administration. However, no changes in food intake and body weight were recorded during the treatment period. Also, plasma cholesterol levels and phytosterol levels, the latter of which strongly depend on nutritional uptake, were unaltered by raised 2-AG levels [27].

In the present study, mice with elevated 2-AG levels displayed an increase in atherosclerotic plaques along with an increase in CD68-positive monocytes and macrophages infiltrating the aortic vessel wall. These data are in keeping with previous work by our group, in which we demonstrated, that a deficient 2-AG biosynthesis diminishes infiltration of the atherosclerotic vessel wall by macrophages and reduces the atherosclerotic plaque burden [15]. An increase in atherosclerotic plaque volume following MAGL-deficiency was also documented by Vujic and coworkers who compared ApoE^-/-^ MAGL^-/-^ double knockout mice to ApoE^-/-^ single knockout controls. In contrast to our work, Vujic et al. reported a non-significant tendency towards a decrease in Mo/Ma-2 positive monocytes and macrophages relative to the atherosclerotic lesions size. This was triggered by the increase in plaque size in ApoE^-/-^ MAGL^-/-^ double knockout mice rather than by a net decrease in Mo/Ma-2 positive cells within the atherosclerotic vessel wall. Intriguingly, Vujic et al. reported increased mRNA levels of CCL2, ICAM-1 and IL-1β within the abdominal aorta of ApoE^-/-^ MAGL^-/-^ double knockout mice which might indicate an elevation in vascular inflammation [16].

For a more detailed characterization of the plaque composition, collagen and smooth muscle cell content were measured within atherosclerotic plaques. While earlier reports by Vujic et al. have found elevated levels of intraplaque smooth muscle cells and an increase in fibrous cap thickness in the aortic sinus, there were no differences in the collagen- and smooth muscle cell content in the present study [16]. However, one has to be cautious when interpreting smooth muscle cell and collagen fiber content in the murine aortic sinus. These data may not be ideal indicators of plaque stability, since murine plaques at this site usually do not rupture (reviewed by [28]). Studies using suitable models like the tandem stenosis model are warranted to assess the influence of 2-AG on plaque stability [29].

Corresponding to our *in vivo* findings, we performed *in vitro* experiments using murine B6MCL macrophages to evaluate the proinflammatory potential of 2-AG. 2-AG has already been shown to enhance monocyte and macrophage migration in several other cellular models including macrophage-like HL-60 cells and human peripheral blood monocytes [14; 30]. In the present study, we showed that murine B6MCL macrophages are equally susceptible to 2-AG induced migration resulting in a migration index that is similar to those reported for other cell models before. Interestingly, we found a marked decrease in macrophage migration following blockade of either CB1 or CB2 receptor, while previous studies have described 2-AG-induced migration to be mainly CB2-dependent. This finding suggests that the CB1 receptor might also be implicated in 2-AG induced macrophage migration, an effect that might add to the proinflammatory potential of CB1 [14; 30]. Intriguingly, preconditioning of B6MCL macrophages with 2-AG was also sufficient to enhance macrophage migration towards FBS-containing culture medium in the present study. 2-AG might enhance macrophage susceptibility for chemokines, possibly by the upregulation of chemokine receptors.

Additionally, 2-AG was shown to impact on adhesion molecules, chemokines and chemokine receptors that are essential to leukocyte recruitment. Our data show an activation of the CCL5-CCR5/CCR1 axis which is implicated in the recruitment of classical monocytes to atherosclerotic lesions [24]. Interestingly, both, CCR1 and CCR5 have been reported to enhance monocyte recruitment. In our experimental setup, treatment with JZL184 did not increase the monocytes counts in the circulation of ApoE^-/-^ mice. This is well in line with findings by Soehnlein et al., who reported CCR1^-/-^ and CCR5^-/-^ mice to display an impaired recruitment of classical monocytes with no effect on classical monocytes counts in the circulation [31]. However, usage of CCL5 as a chemoattractant did not further increase macrophage migration of 2-AG preconditioned macrophages in the present study, arguing that chemokines and chemokine receptors other than the CCL5-CCR5/CCR1 axis also might play a role under the present experimental conditions.

Finally, B6MCL macrophages displayed an increased transcription of scavenger receptor CD36 which mediates cholesterol uptake, following stimulation with 2-AG. Meanwhile, transcription of the cholesterol efflux transporters ABCA1 and ABCG1 was not significantly affected by 2-AG. Similar findings have been reported for murine primary peritoneal macrophages in which the synthetic CB1- and CB2-agonist WIN55,212 led to an increase in the expression of CD36 and to the down-regulation of ABCA1 [32].

Taken together, our data provide evidence for a proinflammatory and atherogenic role of 2-AG both *in vitro* and *in vivo*. Acting via autocrine and paracrine signaling pathways, 2-AG might facilitate leukocyte recruitment to atherosclerotic plaques and thereby contribute to vascular inflammation and atherogenesis. Potential other mechanisms such as reduced macrophage apoptosis in the plaque cannot be excluded. Utilizing the endocannabinoid system for therapeutic purposes is certainly a complicated task, given that the ECS is almost ubiquitously expressed and accomplishes varying functions in different tissues. Targeting the ECS might cause neurological and psychiatric side effects (as seen with the CB1 inverse agonist rimonabant that had to be withdrawn from the market) and might interfere with arachidonic acid, its derivatives and with a variety of lipid signaling molecules that may affect a broad range of diseases. Moreover, apart from the deleterious atherogenic effects reported in this study, genetic and pharmacological inhibition of MAGL have also been associated with a variety of beneficial effects in the treatment of pain, neuroinflammation, neurodegenerative diseases and several cancer entities [23; 33; 34]. These discrepancies between favorable and undesirable effects illustrate that MAGL is an interesting, yet challenging therapeutic target. Clearly, the distinct molecular mechanisms that underlie these effects and the potentially cell-specific characteristics of the ECS and its associated signaling pathways have to be elucidated, before ECS components become suitable drug targets.

## Supporting information

S1 Fig Dose-response curve for JZL184. ApoE^-/-^ mice were treated with increasing concentrations of JZL184 (0.1 mg/kg, 0.5 mg/kg, 5 mg/kg) for one week. Plasma and aortic tissue were collected 24 hours after the last injection. 2-arachidonoylglycerol levels were quantified by liquid chromatography-multiple reaction monitoring. 2-AG, 2-arachidonoylglycerol; JZL184, inhibitor of monoacylglycerol lipase.
(TIF)

S2 Fig Gating strategies for flow cytometry. Leukocytes were stained for CD11b, CD3, CD19, Ly6C, and Ly6G (clones M1/17, 17A2, 1D3, RB6-8C5, AL-21, BD Biosciences, San Jose, USA). Prevalence of these surface markers within a pre-specified leukocyte gate was determined after measuring 50,000 counts. Flow cytometry plots depict the applied gating strategies. CD, cluster of differentiation; IM, inflammatory monocytes; Ly6C, lymphocyte antigen 6C; Ly6G, lymphocyte antigen 6G; NG, neutrophil granulocytes; PM, patrolling monocytes.
(TIF)

S1 Table Antibodies used for histological and flow cytometry staining. All antibodies used for histological stainings and for flow cytometry are listed in this table. α-SMA, α-smooth muscle actin; CD, cluster of differentiation; Ly6C, lymphocyte antigen 6C; Ly6G, lymphocyte antigen 6G. oxLDL, oxidized low-density lipoprotein; UUID, user facing URL resolvable identifier.
(DOCX)

S2 Table TaqMan^®^ probes used for qPCR. This table lists all TaqMan^®^ probes used in this study for qPCR.
(DOCX)

## Abbreviations

2-AG: 2-arachidonoylglycerol
α-SMA: Alpha smooth muscle actin
AA: Arachidonic acid
AEA: N-arachidonoylethanolamide
CB1: Cannabinoid receptor 1
CB2: Cannabinoid receptor 2
CCL5: Chemokine (C-C motif) ligand 5
CCR1: Chemokine (C-C motif) receptor 1
CCR5: Chemokine (C-C motif) receptor 5
DAGLα: Diacylglycerol lipase α
eCBs: Endocannabinoids
LC-MRM: Liquid chromatography-multiple reaction monitoring
MAGL: Monoacylglycerol lipase
PFA: Paraformaldehyde
