# Supplementary figures and images for: Elevated levels of 2-arachidonoylglycerol promote atherogenesis in ApoE-/- mice
Source: PLoS One. 2018 May 29;13(5):e0197751. doi: 10.1371/journal.pone.0197751 (PMC5973571; doi:10.1371/journal.pone.0197751)

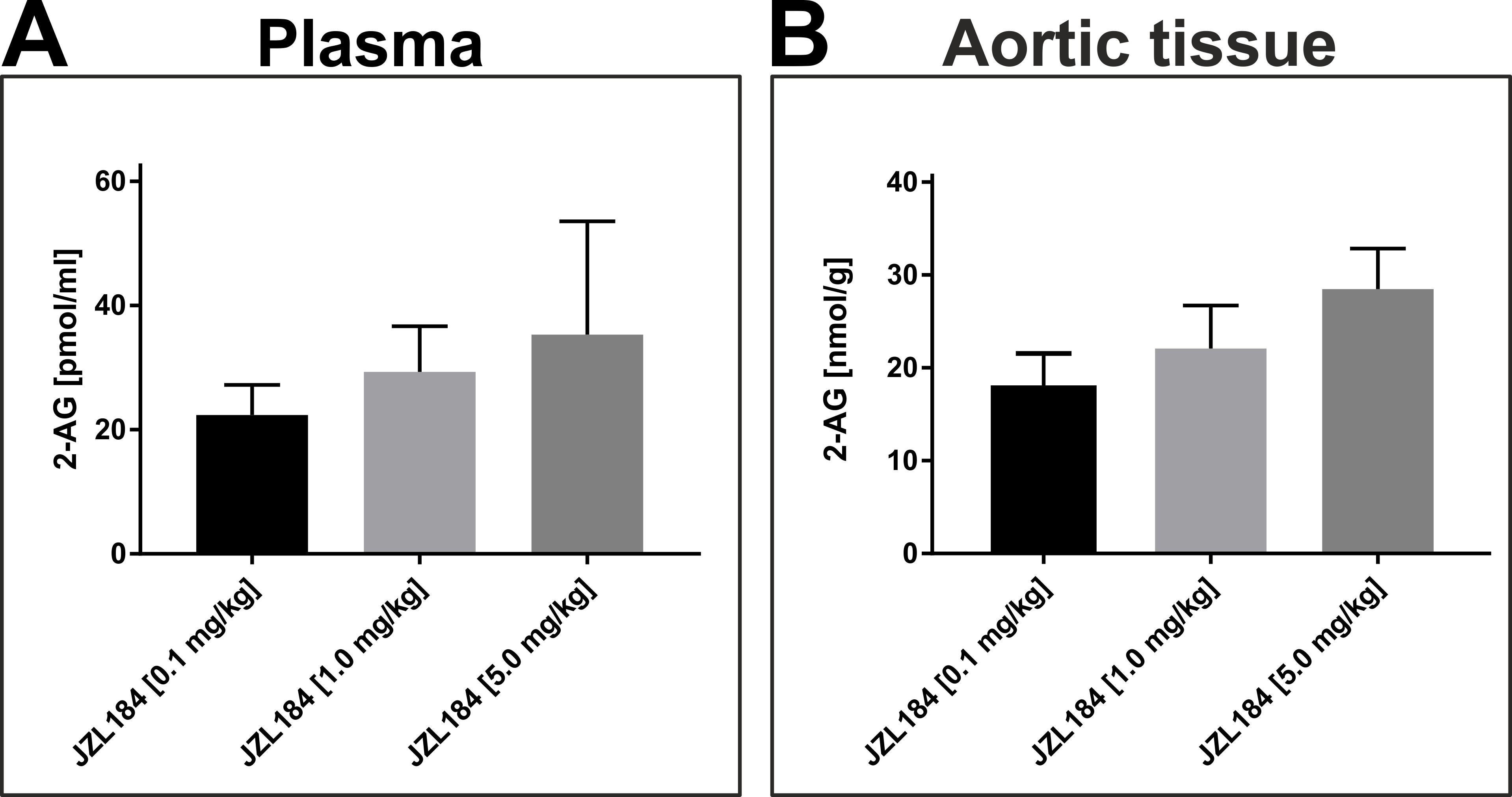

Supplement: S1 Fig — ApoE-/- mice were treated with increasing concentrations of JZL184 (0.1 mg/kg, 0.5 mg/kg, 5 mg/kg) for one week. Plasma and aortic tissue were collected 24 hours after the last injection. 2-arachidonoylglycerol levels were quantified by liquid chromatography-multiple reaction monitoring. 2-AG, 2-arachidonoylglycerol; JZL184, inhibitor of monoacylglycerol lipase. (TIF) [file pone.0197751.s001.tif]

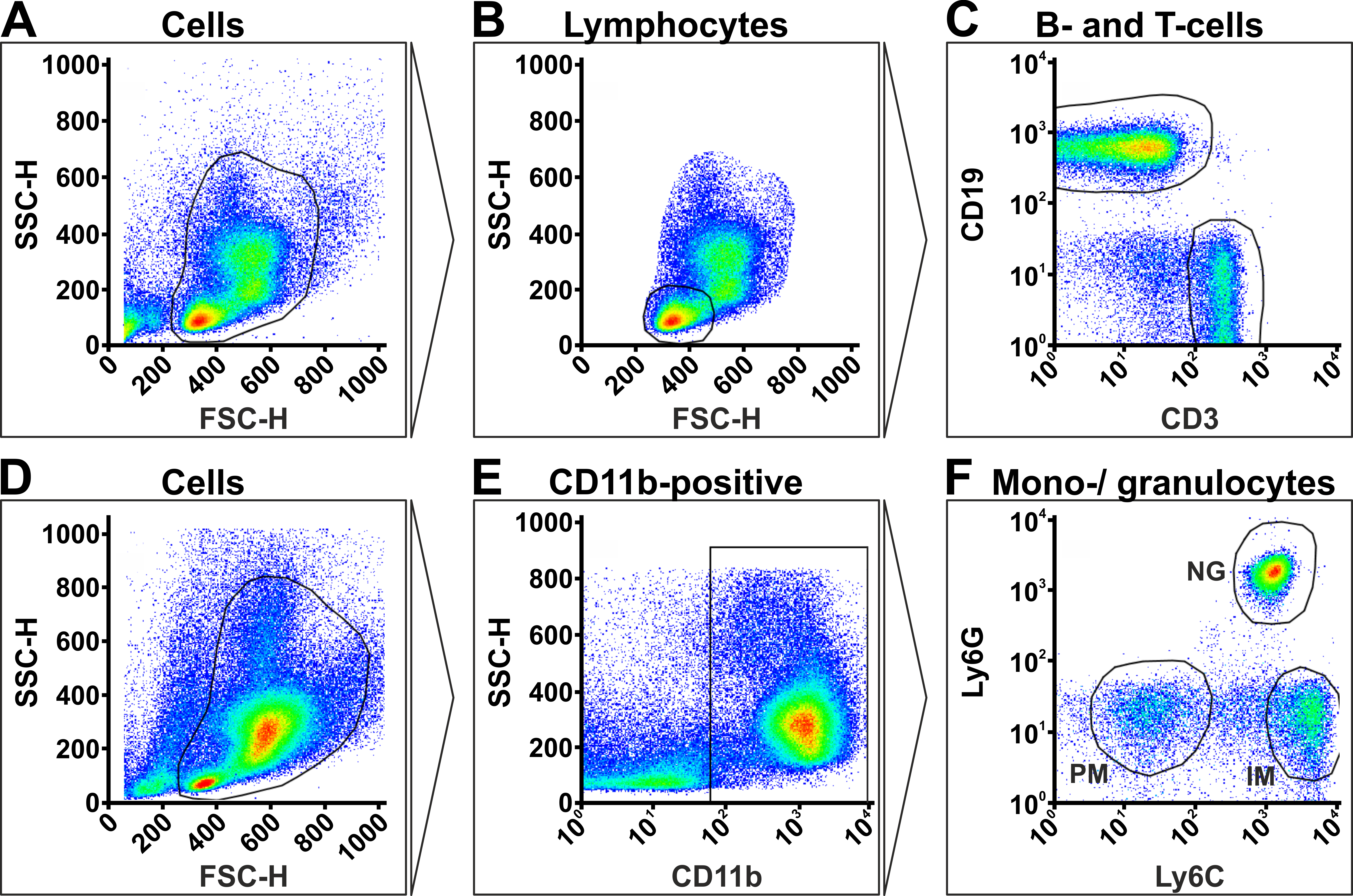

Supplement: S2 Fig — Leukocytes were stained for CD11b, CD3, CD19, Ly6C, and Ly6G (clones M1/17, 17A2, 1D3, RB6-8C5, AL-21, BD Biosciences, San Jose, USA). Prevalence of these surface markers within a pre-specified leukocyte gate was determined after measuring 50,000 counts. Flow cytometry plots depict the applied gating strategies. CD, cluster of differentiation; IM, inflammatory monocytes; Ly6C, lymphocyte antigen 6C; Ly6G, lymphocyte antigen 6G; NG, neutrophil granulocytes; PM, patrolling monocytes. (TIF) [file pone.0197751.s002.tif]
