# Supplementary material for: Elevated levels of 2-arachidonoylglycerol promote atherogenesis in ApoE-/- mice
Source: PLoS One. 2018 May 29;13(5):e0197751. doi: 10.1371/journal.pone.0197751 (PMC5973571; doi:10.1371/journal.pone.0197751)
Supplement: S1 Table — All antibodies used for histological stainings and for flow cytometry are listed in this table. α-SMA, α-smooth muscle actin; CD, cluster of differentiation; Ly6C, lymphocyte antigen 6C; Ly6G, lymphocyte antigen 6G. oxLDL, oxidized low-density lipoprotein; UUID, user facing URL resolvable identifier. (DOCX) [file pone.0197751.s003.docx]

**Supporting information**

**Supplementary Tables:**

**S1 Table: Antibodies used for histological and flow cytometry staining.**

| **AB ID** | **AB Name** | **Target Antigen** | **Vendor** | **Cat Num** | **Proper Citation** | **Reference** | **Clonality** | **Clone ID** | **Host** | **comments** | **V_uuid** |
| --- | --- | --- | --- | --- | --- | --- | --- | --- | --- | --- | --- |
| AB_1001178 | Rat Anti-CD68 (Macrosialin) Monoclonal Antibody, Unconjugated, Clone FA-11 | CD68 (Macrosialin) mouse | Acris Antibodies GmbH | SM1550P | (Acris Antibodies GmbH Cat# SM1550P, RRID:AB_1001178) |  | monoclonal antibody | Clone FA-11 | rat | manufacturer recommendations: Flow Cytometry; Immunohistochemistry; Immunoprecipitation; Western Blot; Immunohistochemistry-frozen, Flow Cytometry, Immunoprecipitation, Western Blot | b4501003-6961-57a4-bca0-6b5cd57c7077 |
| AB_476701 | Mouse Anti-Actin, alpha-Smooth Muscle Monoclonal Antibody, Unconjugated, Clone 1A4 | Actin, alpha, Smooth Muscle bovine, canine, chicken/avian, goat, guinea pig, human, mouse, other, rabbit, rat, sheep, xenopus, human, mouse, rat, bovine, chicken, frog, goat, guinea pig, rabbit, canine, sheep, snake | Sigma-Aldrich | A2547 | (Sigma-Aldrich Cat# A2547, RRID:AB_476701) | PMID:18186028, PMID:27155150, PMID:27257960, PMID:27642710, PMID:28324630, PMID:28340341, PMID:28457748, PMID:28467929, PMID:28590037, PMID:28977592, PMID:28980940 | monoclonal antibody | Clone 1A4 | mouse | Vendor recommendations: Immunofluorescence; Immunohistochemistry; Western Blot; Indirect Immunofluorescence, Immunohistochemistry (Frozen sections), Immunohistochemistry (Paraffin sections), Western Blot | 80c6e7f2-40c7-5bd7-99f5-7a9e4008f3c6 |
| AB_398535 | CD11b antibody | CD11b mouse | BD Biosciences | 553312 | (BD Biosciences Cat# 553312, RRID:AB_398535) | PMID:27638600, PMID:28689984, PMID:28709002, PMID:28886383 | monoclonal antibody | M1/70 | rat | validation status unknown, vendor suggested use: IgG2; IgG2 Flow Cytometry; Flow Cytometry | 292e1ad3-7216-52c1-94f4-7d781549f8fd |
| AB_395698 | Rat Anti-CD3 Molecular Complex Monoclonal Antibody, FITC Conjugated, Clone 17A2 | CD3 Molecular Complex mouse | BD Biosciences | 555274 | (BD Biosciences Cat# 555274, RRID:AB_395698) | PMID:27693350 | monoclonal antibody | 17A2 | rat | validation status unknown, vendor suggested use: Flow Cytometry; FCM | 8546b5b2-6ff0-5084-ad29-1339eab847b1 |
| AB_395050 | Rat Anti-CD19 Monoclonal Antibody, Phycoerythrin Conjugated, Clone 1D3 | CD19 mouse | BD Biosciences | 553786 | (BD Biosciences Cat# 553786, RRID:AB_395050) | PMID:28275164, PMID:28472640, PMID:28559011, PMID:28813662, PMID:29150241 | monoclonal antibody | 1D3 | rat | validation status unknown, vendor suggested use: Flow Cytometry; FCM, FCM*, IC/FCM | 84409f07-e415-5e73-a16e-2e0988bd6981 |
| AB_394628 | Rat Anti-Ly-6C Monoclonal Antibody, FITC Conjugated, Clone AL-21 | Ly-6C mouse | BD Biosciences | 553104 | (BD Biosciences Cat# 553104, RRID:AB_394628) | PMID:27716507, PMID:29126797 | monoclonal antibody | AL-21 | rat | validation status unknown, vendor suggested use: Flow Cytometry; FCM | d0259a7b-0508-50a6-8703-a558229c75d4 |
| AB_394208 | Rat Anti-Ly-6G Monoclonal Antibody, Phycoerythrin Conjugated, Clone 1A8 | Ly-6G mouse | BD Biosciences | 551461 | (BD Biosciences Cat# 551461, RRID:AB_394208) | PMID:28407483, PMID:29150241 | monoclonal antibody | 1A8 | rat | validation status unknown, vendor suggested use: Flow Cytometry; Immunohistochemistry; Immunoprecipitation; FCM, IHC(F), IHC(Fr), IHC(Zn), IP | 018171ca-1e85-5f5b-bca7-5c9e7073b3c8 |
| AB_2340667 | Cy3-AffiniPure Donkey Anti-Rat IgG (H+L) (min X Bov,Ck,Gt,GP,Sy Hms,Hrs,Hu,Ms,Rb,Shp Sr Prot) antibody | Rat IgG (H+L) | Jackson ImmunoResearch Labs | 712-165-153 | (Jackson ImmunoResearch Labs Cat# 712-165-153, RRID:AB_2340667) | PMID:28111078, PMID:28245922, PMID:28340341, PMID:28502656, PMID:28583742, PMID:28602823, PMID:28669757, PMID:28781050, PMID:28957665 | polyclonal antibody |  |  | Originating manufacturer of this product | d7b79745-1bfd-5f81-8f2c-2fa741a09c08 |
