# Supplementary material for: Elevated levels of 2-arachidonoylglycerol promote atherogenesis in ApoE-/- mice
Source: PLoS One. 2018 May 29;13(5):e0197751. doi: 10.1371/journal.pone.0197751 (PMC5973571; doi:10.1371/journal.pone.0197751)
Supplement: S2 Table — This table lists all TaqMan® probes used in this study for qPCR. (DOCX) [file pone.0197751.s004.docx]

**Supporting information**

**Supplementary Tables:**

**S2 Table: TaqMan® probes used for qPCR.**

| **TaqMan® Gene Expression Assay ID** | **Target Gene** | **Vendor** | **Cat Num** |
| --- | --- | --- | --- |
| Mm01216147_m1 | Ccr1  chemokine (C-C motif) receptor 1 | Thermo Fisher Scientific Inc. | 4331182 |
| Mm00516023_m1 | Icam1  intercellular adhesion molecule 1 | Thermo Fisher Scientific Inc. | 4331182 |
| Mm01302427_m1 | Ccl5  chemokine (C-C motif) ligand 5 | Thermo Fisher Scientific Inc. | 4331182 |
| Mm01216171_m1 | Ccr5  chemokine (C-C motif) receptor 5 | Thermo Fisher Scientific Inc. | 4331182 |
| Mm00441242_m1 | Ccl2  chemokine (C-C motif) ligand 2 | Thermo Fisher Scientific Inc. | 4331182 |
| Mm01320970_m1 | Vcam1  vascular cell adhesion molecule 1 | Thermo Fisher Scientific Inc. | 4331182 |
| Mm00442646_m1 | Abca1  ATP binding cassette subfamily A member 1 | Thermo Fisher Scientific Inc. | 4331182 |
| Mm00437390_m1 | Abcg1  ATP Binding Cassette Subfamily G Member 1 | Thermo Fisher Scientific Inc. | 4331182 |
| Mm01135198_m1 | CD36  cluster of differentiation 36 | Thermo Fisher Scientific Inc. | 4331182 |
